# Predicting technological innovation in new energy vehicles based on an improved radial basis function neural network for policy synergy

**DOI:** 10.1371/journal.pone.0271316

**Published:** 2022-08-25

**Authors:** Ying Hao, Mingshun Guo, Yijing Guo

**Affiliations:** 1 School of Management, Shenyang University of Technology, Shenyang, China; 2 Dean’s Office, Liaoning Engineering Vocational College, Tieling, China; National Institute of Technology Silchar, India, INDIA

## Abstract

Policy synergy is necessary to promote technological innovation and sustainable industrial development. A radial basis function (RBF) neural network model with an automatic coding machine and fractional momentum was proposed for the prediction of technological innovation. Policy keywords for China’s new energy vehicle policies issued over the years were quantified by the use of an Latent Dirichlet Allocation (LDA) model. The training of the neural network model was completed by using policy keywords, synergy was measured as the input layer, and the number of synchronous patent applications was measured as the output layer. The predictive efficacies of the traditional neural network model and the improved neural network model were compared again to verify the applicability and accuracy of the improved neural network. Finally, the influence of the degree of synergy on technological innovation was revealed by changing the intensity of policy measures. This study provides a basis for the relevant departments to formulate industrial policies and improve innovation performance by enterprises.

## Introduction

There is a close relationship between innovation by enterprises and the environment [1]. Innovation is influenced by national policies and support systems [2]. Policy synergy refers to the mutual coordination and cooperation of different policy measures by relevant government departments to generate policy synergy that promotes the realization of policy objectives [3]. Relying on the optimization of the policy structure, certain system functions can be achieved by coupling the policy functions [4]. As a strategic emerging industry, the capability for NEV technological innovation is influenced by guidance and support from industrial policies. In quite a long period of time, the development of the NEV industry has been affected by various policies and its policy-driven characteristics have been obvious. Policy has become the key to technological innovation by NEV enterprises. A high complex policy space is formed by each policies [5]. For example, the improvement of technical standards driven by subsidy policy can more effectively promote enterprise technological innovation, and the infrastructure construction guided by policy can reduce the market risk of enterprise technological innovation. The environment of technological innovation of new energy vehicle enterprises is constituted by the synergy promotion of various policy measures [6]. The synergy of different policy measures has different impacts on the technological innovation of new energy vehicles [7]. The government should actively adjust the policy structure [8] and implement different policy synergy models [9, 10] to effectively stimulate technological innovation and promote sustainable industrial development [11] for further integration of industry and policy.

Accurate prediction of technological innovation can provide scientific guidance for enterprises to make development decisions [12] and improve comprehensive competitiveness [13]. Patents contain the details of technological innovation, and the development trend of technological innovation can be grasped through scientific analysis of patent information, which is an effective tool for innovation technology prediction [14]. Traditional innovation predictions have usually applied regression analysis [15], time series analysis [16, 17], Markov chains [18], DEA efficiency models [19], and comprehensive control methods [20]. As a typical representative of an intelligent prediction model, neural network can mine complex nonlinear features behind data without assuming the relationships between variables and has a strong generalization ability [21, 22]. Radical Basic Function (RBF) neural network is a three-layer feedforward analysis network, which has strong robustness, self-learning ability and nonlinear mapping ability. The structure parameters can achieve separation of learning and the training speed is fast because of the compact topology. The radial basis function is used to control the hidden layer nodes and carry out high-dimensional nonlinear transformation, which can approximate any nonlinear function. It is a relatively ideal nonlinear calculation model and has been widely used in technological innovation research [23]. With the deepening of research, cloud model [23], gray GM(1,1) model [24] and intelligent algorithm [25] are also used for the improvement of RBF neural network model.

RBF neural network model is introduced to predict the technological innovation of new energy vehicle enterprises in this paper. An improved RBF neural network model based on automatic coding machine is proposed, and a fractional momentum algorithm is designed. On the basis of ensuring the prediction accuracy and stability of the model, the prediction of the technological innovation of new energy vehicle enterprises for policy synergy is realized. Based on the simulation results of the proposed model, the applicable conditions of policy synergy under different situations were analyzed to assist relevant government departments to formulate effective industrial policies and stimulate innovation. At the same time, it provides reference for enterprises to make accurate innovation decisions.

## Research design

### Research process

**Fig 1:**
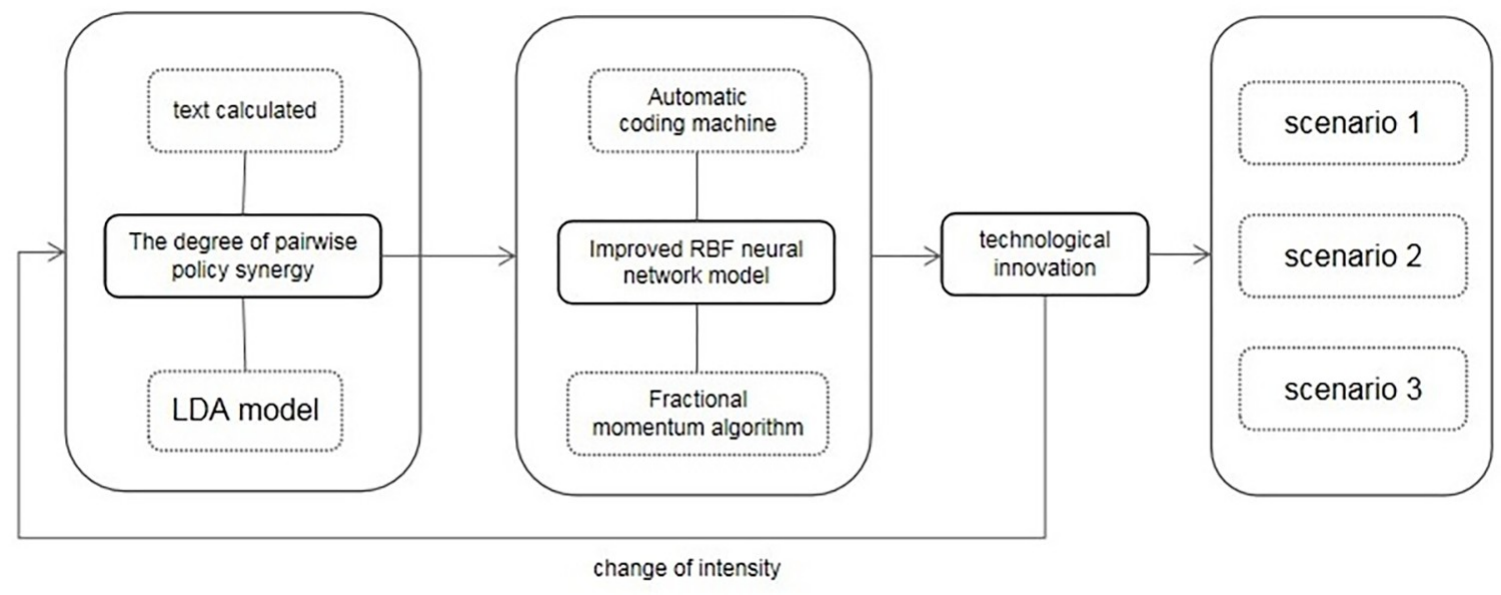
Research process.

Policy synergy is the core influencing factor of technological innovation of new energy vehicle enterprises. In order to analyze the relationship between the two, the RBF neural network model is improved to adapt to the high coupling of multiple inputs. The model is trained with the degree of policy synergy as input and the number of valid patents applied by enterprises as output. Finally, by changing the policy intensity to adjust the degree of synergy, the model is used to predict the number of patents. The specific process is shown in Fig 1.

#### Construction of the model

The degrees of the synergy of different policy measures of NEVs have different effects, mechanisms, and directions. with a complex nonlinear relationship among them for technological innovation. The synergy between different policy measures affects each other and there is a complex coupling relationship. The nonlinear mapping between the degree of policy synergy and technological innovation was taken as the training samples used in this study. To establish a high-precision and high-stability predictive model, a neural network with strong adaptability was required. We made adaptive improvements to the RBF neural network in accordance with the descriptions of the characteristics of the training samples. Table 1 shows the specific problem descriptions and their corresponding solutions.

**Table 1:** Problem descriptions and solutions.

	Problem description	Solution
1	There is a highly coupled relationship between degree of policy synergy in the sample input data. It is necessary to extract the main features of the sample data keenly.	An automatic coding machine is a type of unsupervised neural network model, which not only can achieve feature dimension reduction but also has the function of feature extraction. Therefore, a hybrid RBF neural network model with an automatic coding machine was designed. The automatic coding machine was adjusted for targeted feature extraction.
2	The traditional gradient algorithm cannot guarantee accurate predictions of technological innovation because it is easy to fall into the local optimal value. The direction of decision-making is determined by accurate predictions of technological innovation and needs the guarantee of a high-precision, high-stability, and comprehensive calculation method.	Since fractional order can improve the momentum memorability of network weights in the optimization process, a fractional momentum RBF neural network model was designed.

The main innovations of the model are as follows.

•An interpretable automatic coding machine that quantifies the importance of input data, extracts features, and suppresses interference features.

•A fractional momentum RBF neural network that improves the memory capacity of the model to realize the effective learning of important features and make accurate predictions of patent information.

#### Structure of proposed model

**Fig 2:**
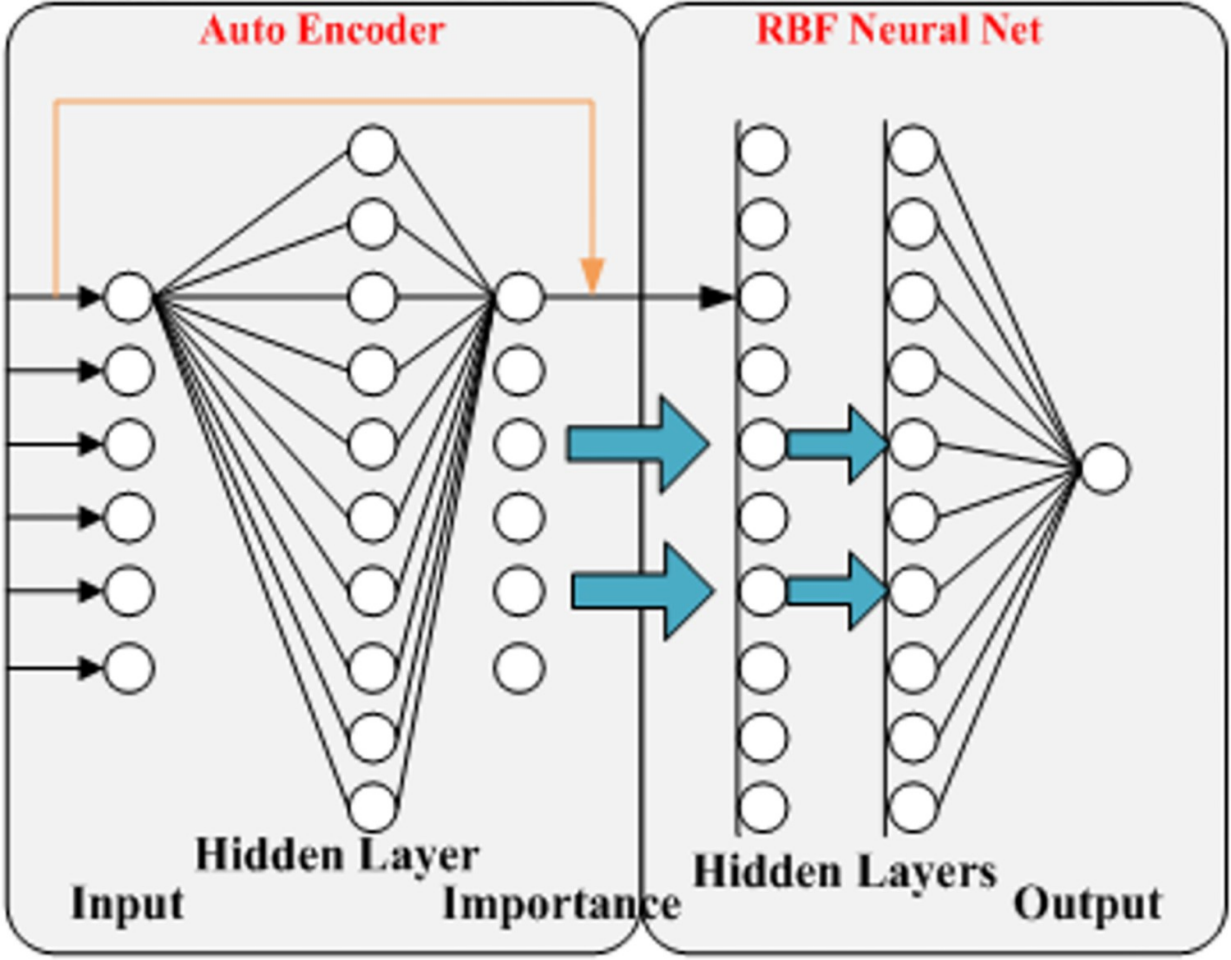
Structure of proposed model.

The structure of the model proposed in this study is shown in Fig 2. The model consists of an automatic coding machine and an RBF neural network. The input layer of the automatic coder receives policy information, which contains six variables. The hidden layer contains 10 neurons for unsupervised training and the output layer contains 6 neurons, representing the importance of the six input variables. The variables in the input layer are weighted and multiplied by their corresponding importance, then the results are passed to the RBF neural network as input data. The network adopts a double hidden layer structure, with each hidden layer containing 10 neurons for feature learning and 1 output layer neuron representing the predicted value of the number of patent applications under this policy.

#### Feature importance analysis algorithm for automatic coding machine

An automatic coding machine is composed of an input layer and a hidden layer. A two-way full connection is used between the input layer and hidden layer; however, there is no connection within the same layer. Eq (1) denotes the value of the hidden layer node obtained from the known input layer node:

$$p(h_{j}=1)=\frac{1}{1+\exp\left(-b_{j}-\sum_{i}v_{i}w_{ij}\right)}$$ (1)

Because the automatic encoder is a symmetric network, the value of the input layer node can be calculated from the hidden layer node in the backpropagation, as shown in Eq (2):

$$p(v_{i}=1)=\frac{1}{1+exp(-c_{i}-\sum_{j}h_{j}w_{ji})}$$ (2)

where *ν*_*i*_ is the value of the node *i* in the input layer; *h*_*j*_ denotes the value of node *j* in the hidden layer; *b* and *c* represent the offset values of the input layer and the hidden layer respectively; *w*_*ij*_ is the weight of the nodes in the input layer and the hidden layer.

An automatic encoding machine adopts unsupervised training for feature extraction. In the process of network training, Eqs (3)–(4) are used to update the weight:

$$w^{\left(\tau +1\right)}=w^{\left(\tau \right)}+\eta \frac{\partial logP(v,h)}{\partial w}$$ (3)

$$\frac{\partial logP(v,h)}{\partial w_{ij}}=<h_{j}^{0}v_{i}^{0}>-<h_{j}^{1}v_{i}^{1}>$$ (4)

where *η* is the learning rate, which affects learning progress. An appropriate learning rate is necessary to ensure the best learning status of weights. $v_{i}^{m}$ represents the feature vector of neuron *i* at *instant t = m*. For example, *ν*^*0*^ is the feature vector when *t = 0*; *h*^*0*^ denotes the feature vector of the hidden layer in Eq (1) and *ν*^*1*^ is the feature vector when *t = 1* in Eq (2). <h^0^v^0^> and <h^1^v^1^> are the mean values of the product of the input feature vectors and their corresponding hidden layer feature vectors.

The input data of the model is the degree of synergy between the two policy measures. There is a competitive relationship between the degree of policy synergy and the two policy measures, which results in complex, nonlinear and mutual coupling between the input variables; thus, each group of data does not exist independently. To predict the number of patent applications accurately and achieve targeted training of the neural network, it is necessary to quantify the importance of input variables and achieve targeted training of neural networks. This paper proposes the use of the following formula to calculate the importance of the input variables in the unsupervised training process of an automatic coding machine:

$${FI}_{i}^{unsup}=\frac{\sum w_{i}^{0}-\sum w_{i}^{n}}{H}$$ (5)

$$I_{i}={v_{i}\cdot FI}_{i}^{unsup}$$ (6)

where ${FI}_{i}^{unsup}$ is the importance of variable i in unsupervised learning, and $w_{i}^{n}$ denotes the weight of variable i in the n-iteration process. *H* is the number of neurons in the hidden layer and *I*_*i*_ represents the integrated input value of neuron i of the RBF neural network obtained by calculation.

The specific process of feature extraction is as follows:

Step 1: The policy synergy variables are input in the input layer.
Step 2: The importance of the policy synergy degree is calculated. And the hidden layer *p(h*_*j*_*)* is achieved through the nonlinear function mapping of weight ω_*ij*_ and bias value *b*_*j*_ in the coding layer.
Step 3: The comprehensive parameter of importance of policy synergy degree is calculated by Formula (6). The output layer p(v_i_) is obtained through the weight ω_*ij*_ *and* bais value *b*_*i*_ back-project the hidden layer.

#### RBF neural network with fractional momentum

The RBF network is a typical three-layer feed-forward neural network that consists of an input layer, hidden layer, and output layer. The input layer receives information from the outside world and passes it to the hidden layer, which integrates and maps the received information. The output layer is a linear layer that responds to the activation signals acting on it. For this study, the RBF network structure was set to 6-10-10-1 (6 input nodes, 10–10 hidden layer nodes, and 1 output node, respectively). The hidden layer transfer function is the radial basis function, and the standard Gaussian function is often expressed, thus:

$$\Phi_{j}(x)=e^{-\frac{{\left\|x-c_{j}\right\|}^{2}}{2\sigma_{j}^{2}}}$$ (7)

where *c*_*j*_ is the center vector of node *j and σ*_*j*_ denotes the width of node *j*. Then, the output value of the network output layer is,

y=w·Φ (8)

For the reverse calculation, a fractional momentum algorithm was designed to improve the traditional stochastic gradient descent method in order to obtain a faster and more stable convergence effect for the model. The following formula is used for the iterative update of the weight:

$$w(k)=w(k-1)+\sum_{q=1}^{k}{\left(-1\right)}^{q+1}\left(\begin{array}{c} \gamma \\ q \end{array}\right)v(k-q)-\eta \frac{\partial E}{\partial w(k-1)}$$ (9)

$$\left(\begin{array}{c} \gamma \\ q \end{array}\right)=\left\{ \begin{array}{l} \frac{\gamma (\gamma -1)\cdot \cdot \cdot (\gamma -q+1)}{q!},\;q\geq 1\\ 1,\;q=0 \end{array}\right.$$

$$v(k)=\mu v(k-1)-\eta \frac{\partial E}{\partial w(k-1)}$$

$$\gamma \in [\gamma_{min},\gamma_{max}]$$

$$\gamma =\left(\frac{k-1}{M-1}\right)(\gamma_{max}-\gamma_{min})+\gamma_{min}$$

where *w* is the weight value, *k* represents the number of iterations, *η* is the learning rate, *γ* is the fractional order, $\left(\begin{array}{c} \gamma \\ q \end{array}\right)$ denotes the generalized binomial coefficient for fractional difference operations, *E* is the objective function, $\frac{\partial E}{\partial w(k-1)}$ is the gradient by iteration, *ν* is the momentum, and *μ* is the momentum factor.

#### Training steps

An RBF neural network model with an automatic coding machine and fractional momentum was proposed. The specific calculation steps are as follows.

Step 1: the network is randomly initialized and six neurons in the input layer receive six input variables.
Step 2: an automatic coding machine for unsupervised training executes Eqs (1)–(2) and the weight is updated by Eqs (3)–(4).
Step 3: the characteristic importance of each input variable is calculated by Eq (5).
Step 4: Eq (6) is used to calculate the comprehensive parameters of the importance of variables, which are the input of the RBF neural network.
Step 5: the forward operation process of the RBF neural network is calculated by Eqs (7)–(8).
Step 6: Eq (9) is used to calculate the reverse operation process of the RBF through the fractional momentum algorithm and the weight is updated.
Step 7: the training of the whole model is completed.

### Selection and description of index

#### Source of data

Policy documents from 2012 to 2020 related to the national NEV industry are collected from the Database of the China Automobile Industry Information Network, Peking University Legal Information Network, websites of relevant government departments, “Energy-saving and NEV Yearbooks”, “China Auto Market Yearbook”, etc. as the input layer data. 145 are finally identified for the policy research database used by this study through the verification and screening one by one.

Take the Patent Retrieval and Analysis database of the State Intellectual Property Office of the People’s Republic of China as the source. The patent data for which the applicant is "company" or "factory" is screened.

#### Input layer data of improved neural network model

The degree of pairwise policy synergy is taken as the input layer data. The calculation process of synergy degree is as follows:

Step 1: The effective policies in the current quarter are encoded. LDA model is used to cluster the keywords and determine the policy category of new energy vehicles.
Step 2: The parameter training and theme extraction of the pre-processed new energy vehicle policy text are carried out to calculate the theme intensity; Finally, according to the content of keywords under each theme, it can be classified into four categories: planning and guiding measures, infrastructure construction measures, technical standard measures and fiscal and tax measures.
Step 3: The average synergy degree of pairwise policies and measures of new energy vehicles in each quarter is calculated by the formula [26].

$$APC_{ts}=\frac{\sum pe_{j}\times pm_{j}^{x}\times pm_{j}^{y}}{N}\;x\neq y,t\in [2012,2020]$$ (10)

where *APCts* is the average policy synergy in quarter *s* of year *t*. *pe*_*j*_ denotes the intensity of policy *j*, which is the product of policies *x* and *y*. Policy intensity refers to the relative proportion of each topic out of all the effective policies in the corpus for each quarter:

$$p_{m}=\frac{\sum_{m}^{k_{i}}\theta_{m}{}_{p}}{z_{p}}\;(m=1,2,\ldots ,i),\;t\in [2012,2020],\;s\in [1,4]$$ (11)

where *pm*_*j*_^*x*^ and *pm*_*j*_^*y*^ represent the scores of policy measures *x* and *y* in policy *j* respectively, which are calculated from the product of the intensity and frequency of the occurrence of each subject word in each policy. *x* and *y* are the two fiscal and tax policy measures, technical standard measures, planning and guiding measures, and infrastructure construction measures.

**Fig 3:**
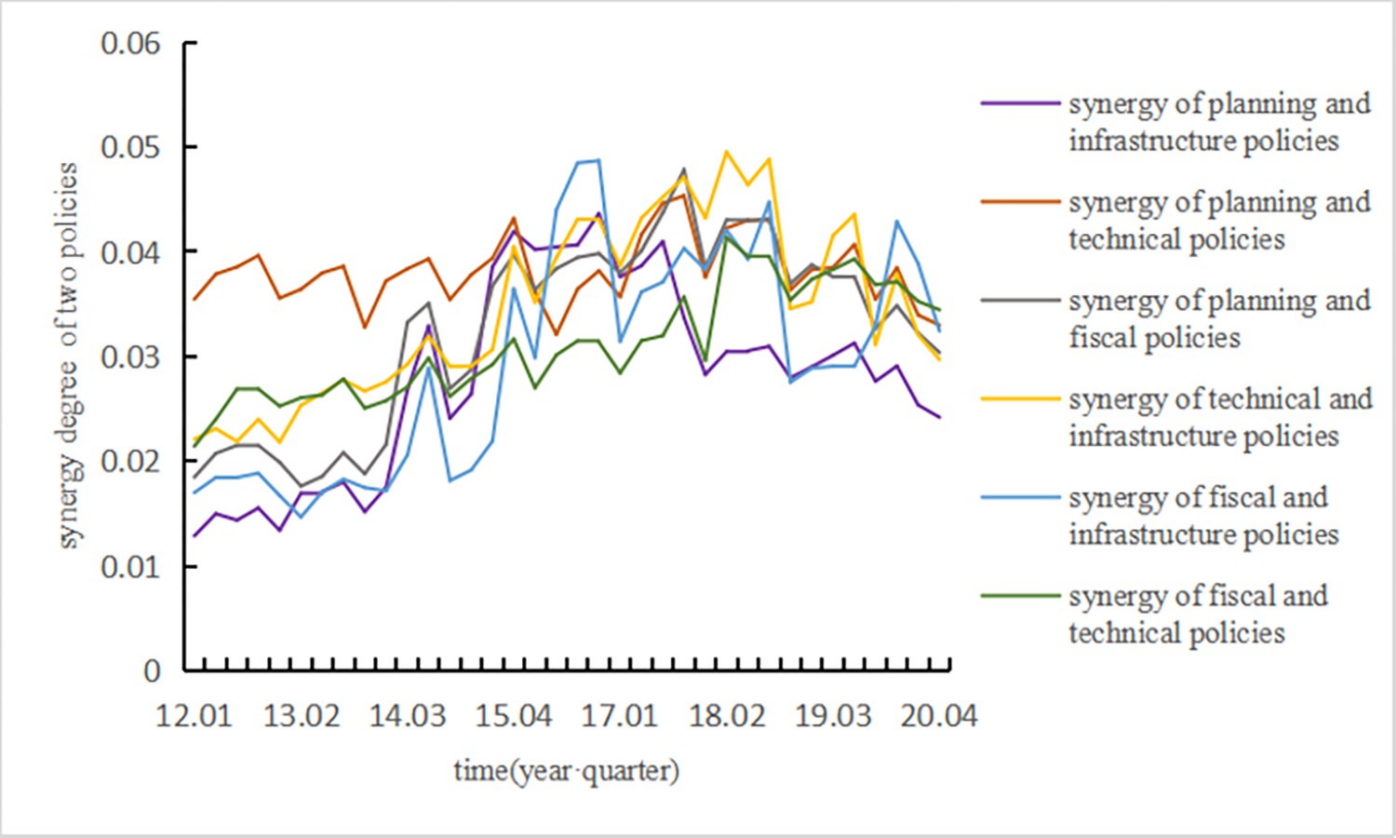
Policy synergy in each quarter from 2012 to 2020.

The input layer data can be described by the calculation of the degree of synergy, as shown in Fig 3.

#### Output layer data of improved neural network model

**Fig 4:**
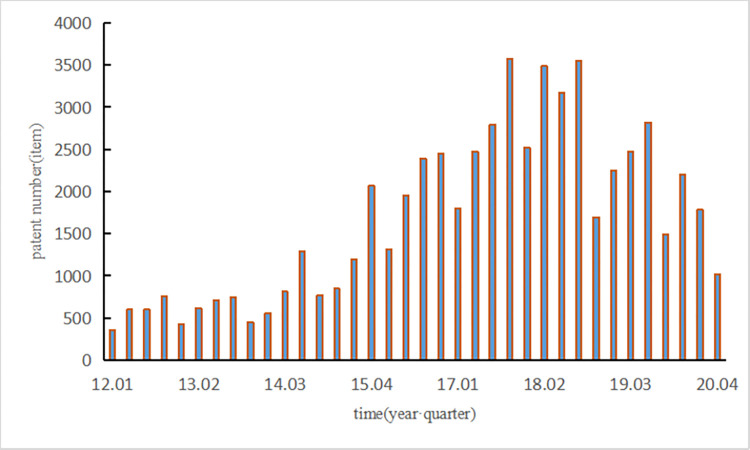
Number of patent applications in each quarter from 2012 to 2020.

The combined retrieval of technologies distributed in different patent categories by defining keywords can overcome the omission issues caused by disputes over the classification numbers of the IPC method and can ensure the directivity of patent information [27–29]. “NEVs”, “electric vehicles”, “fuel cell vehicles”, and “hybrid electric vehicles” were used as the keywords [30], a total of 59,962 patents were retrieved by a screening of the patent data. The output layer data are presented in Fig 4.

## Empirical results and analysis

### Prediction results of the improved neural network model

In the training of the proposed model, the set parameters included the structural parameters and training parameters of the neural network. The structural parameters included the number of hidden layers while the number of neurons and training parameters included training step size, number of iterations, number of important data for the automatic coding machine, and the fractional momentum value of the RBF network. In this experiment, the number of hidden layers of the automatic coding machine was 1, the number of hidden layers was 20, the number of the hidden layers of the RBF network was 2, and the number of neurons in each layer was 10. The training step was set to 0.01, the number of iterations for the automatic coder was set to 20, and the number of iterations for the RBF network was set to 100 (or reached the training condition to stop in case of an error). It showed that all the features played a role in model prediction from the experimental analysis. The importance of the input data from the six features was significantly greater than 0. Therefore, all the six neurons in the input layer were retained and the follow-up experiment was completed.

**Fig 5:**
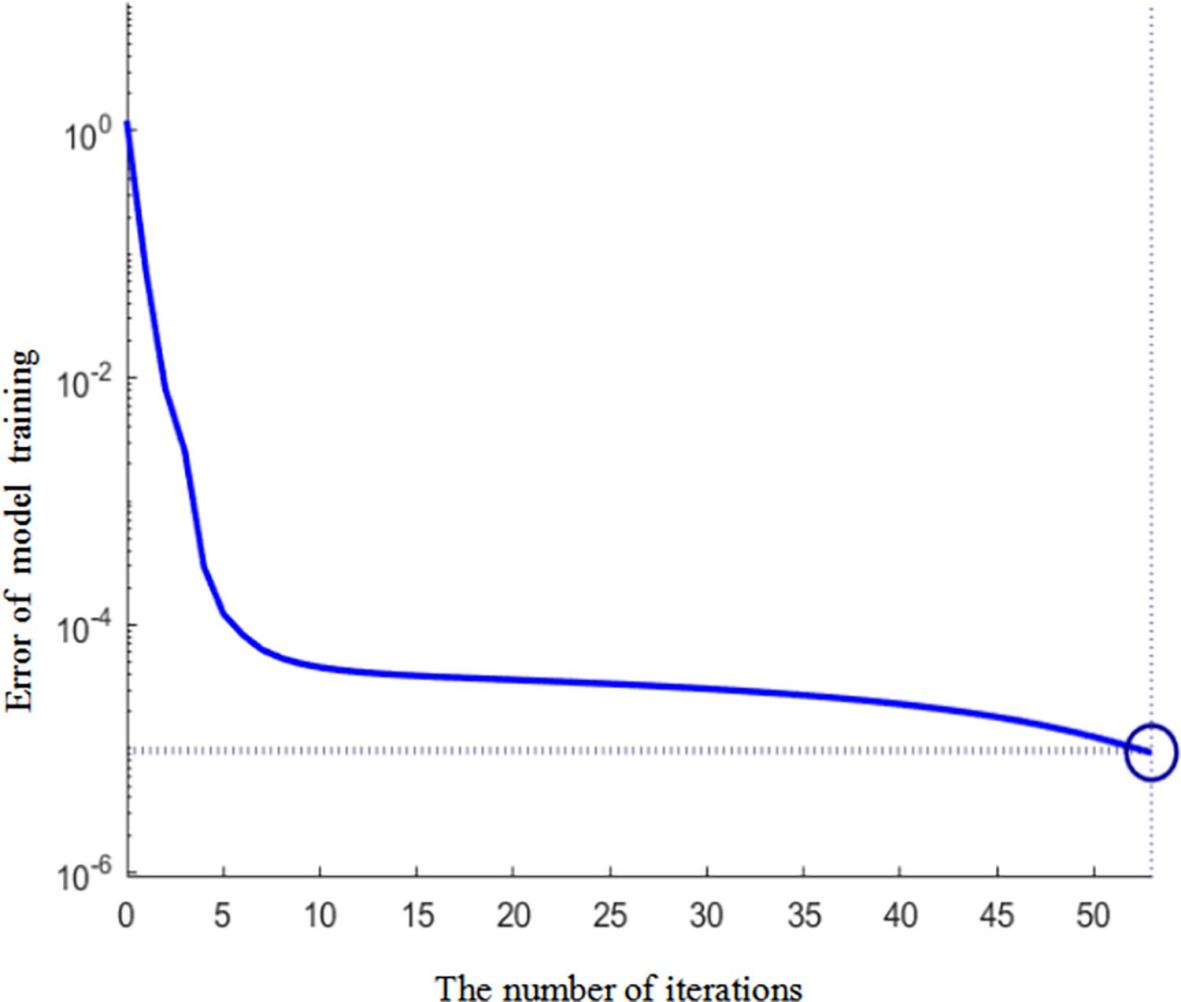
Model training iteration diagram.

Next, the degree of the policy synergy between two policies was treated as the input data and the number of synchronous patent applications was treated as the output data. There was a total of 36 groups of experimental data corresponding to each quarter from 2012 to 2020. The first 32 groups were used to complete the training of the improved RBF neural network model and the last 4 groups of data were used as the test data. Fig 5 is the iterative diagram of the model training.

To further verify the advantages of the model proposed in this paper, especially the designed interpretable automatic coder and fractional momentum RBF neural network model, the training speed and training accuracy were compared with those of the BP model. Table 2 shows a comparison of the errors produced by both models.

**Table 2:** Comparison of errors between different models.

	Number of training iterations	Average error
Our model	54	7.65%
RBFmodel	87	9.46%
BP-ANN	122	10.57%
Automatic coding machine	95	10.23%
SVM	114	9.81%
DBN	98	8.79%

Table 2 shows that: (1) The number of iterations of our model are significantly less than those of other neural network models and indicates that the interpretable automatic coder could realize the analysis of the importance of the data, effectively remove interference features, and improve the learning effects of the useful features. Also, the interpretable automatic coder improved the calculation efficiency of the automatic coder, thus reducing the costs and improving the results of the calculations. The interpretability and readability of the model have been improved and the results of the training effect have met expectations. (2) The errors of our model are significantly smaller than those of other neural network models, indicating that the network weight could make use of the momentum memory in the optimization instead of relying only on the momentum obtained in the last iteration. Hence, our model can make the updating of the weights more stable. The results of the training have shown that our model is more adaptive to predictions of technological innovation by NEVs.

### Stability analysis of improved neural network model

To verify the stability of our model in predicting technological innovation by applying the degree of synergy, Eq (10) is improved to measure the degree of the synergy among three policies:

$$APC_{ts}=\frac{\sum pe_{j}\times pm_{j}^{x}\times pm_{j}^{y}\times pm_{j}^{z}}{N}\;x\neq y\neq z,t\in [2012,2020]$$ (12)

**Fig 6:**
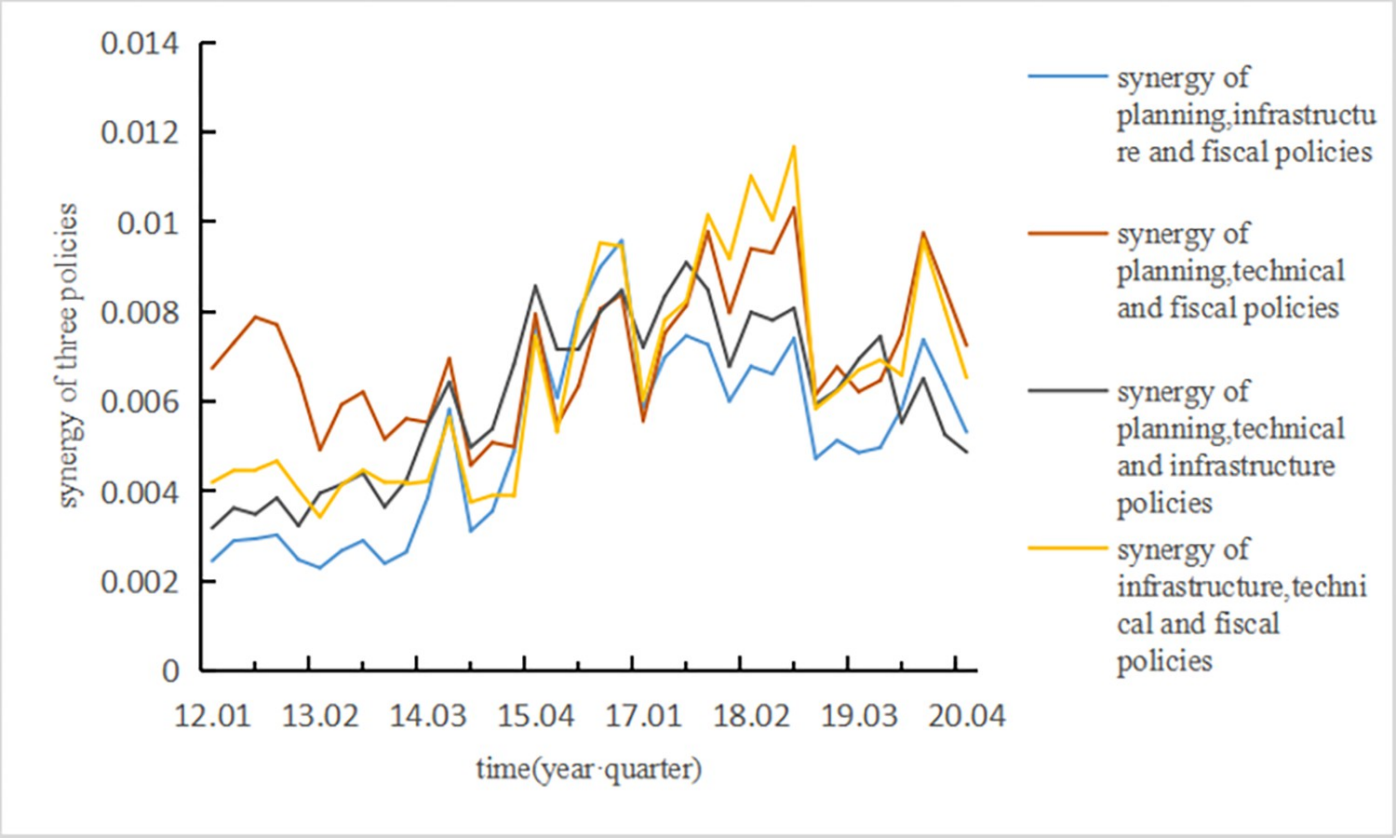
Synergy among three policy measures in each quarter from 2012 to 2020.

The calculation results of the degree of synergy among the three policies are shown in Fig 6. The experiment described in Section 3.1 was repeated. To train our model, the degree of the synergy among the three policies was treated as the input layer data while the application technological innovation data of each quarter from 2012 to 2020 in Fig 4 was treated as the output layer. Finally, the results of the last four groups of data show that the number of iterations is 52 and the average error is 7.82%, thus indicating that our model has high stability. Meanwhile, the experiment has shown that our model is more accurate at predicting technological innovation with the synergy between two policies as the input layer. Then, we analyzed the influence law of the technological innovation states of NEV enterprises.

### Influence law of synergy of policy measures on technological innovation by NEVs

To further analyze the effects of the degree of the synergy among the policy measures on technological innovation by NEV enterprises, the adjustment of the intensity of the policy measures was used to explore the effects of the policies and to measure the change in the degree of synergy. On the basis of the calculation method for two degrees of policy synergy, the change in the intensity of single policy measures, two simultaneous policy measures, and three policy measures were adjusted. Eq (10) was used to retrieve the input data of the model and complete the technological innovation state prediction of NEV enterprises under corresponding conditions. On this basis, different forms of effective policy synergy to stimulate innovation are put forward.

Scenario 1: The influence rule of the technological innovation state of NEV enterprises when the intensity of single policy measure increase 5%, 10%, 15%, and 20% respectively.

**Fig 7:**
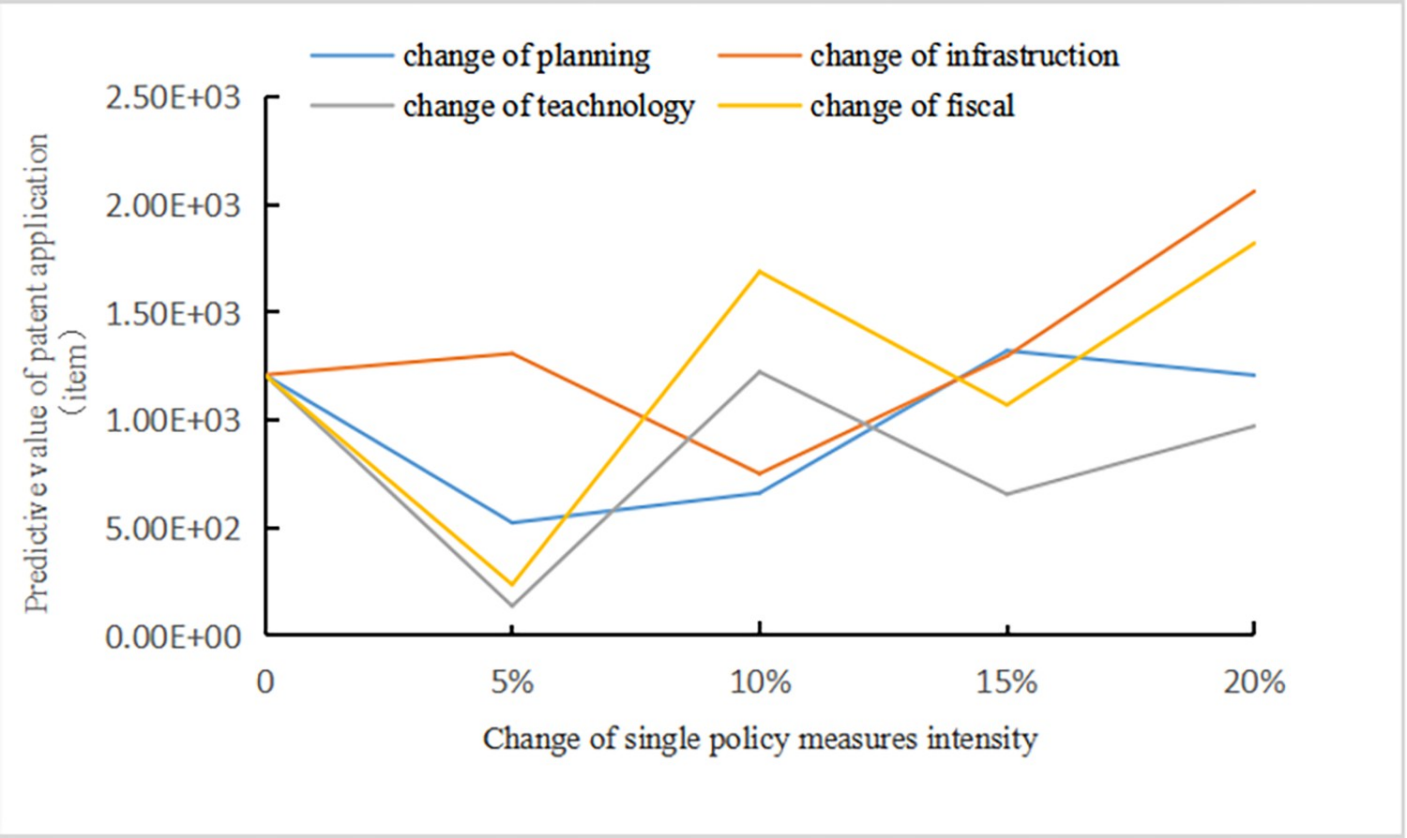
Relationship between intensity of individual policy measures and number of patent applications.

Fig 7 shows that with the increase of single policy measure intensity, the overall trend of enterprise technological innovation is on the rise. The comparison of the effects of increases in the intensity of single policy measures on the number of patent applications has shown that increases in the intensity of infrastructure construction and tax policy measures have had stronger incentive effects on the number of patent applications. The impact of intensity change of planning guidance measures on technological innovation is relatively small. A linear increase in the number of patent applications has not been achieved by changing the intensity of a single policy measure. Under the coupling effects of different policy measures, technological innovation by NEV enterprises presents an unbalanced and inconsistent phenomenon. Even under the influence of the degree of synergy between policies, an increase in the intensity of single policy measures will reduce the number of patent applications and restrict technological innovation by NEV enterprises.

This scenario applies to the very early stage of the development of the new energy vehicle industry when the policy system is not perfect. A significant increase in the intensity of single policy measure can promote technological innovation, which can be maintained for an appropriate time. By increasing the intensity of infrastructure or fiscal and tax policy measure, the incentive effect of innovation can be maximized. And then the strategic layout of driving industrial development by innovation can be formed. However, increasing the policy intensity of planning and guiding measures alone without the synergy of other policies will restrict the technological innovation of enterprises.

Scenario 2:The influence rule of the technological innovation states of NEV enterprises when the intensity of the two policy measures increase by 5%, 10%, 15%, and 20% simultaneously.

**Fig 8:**
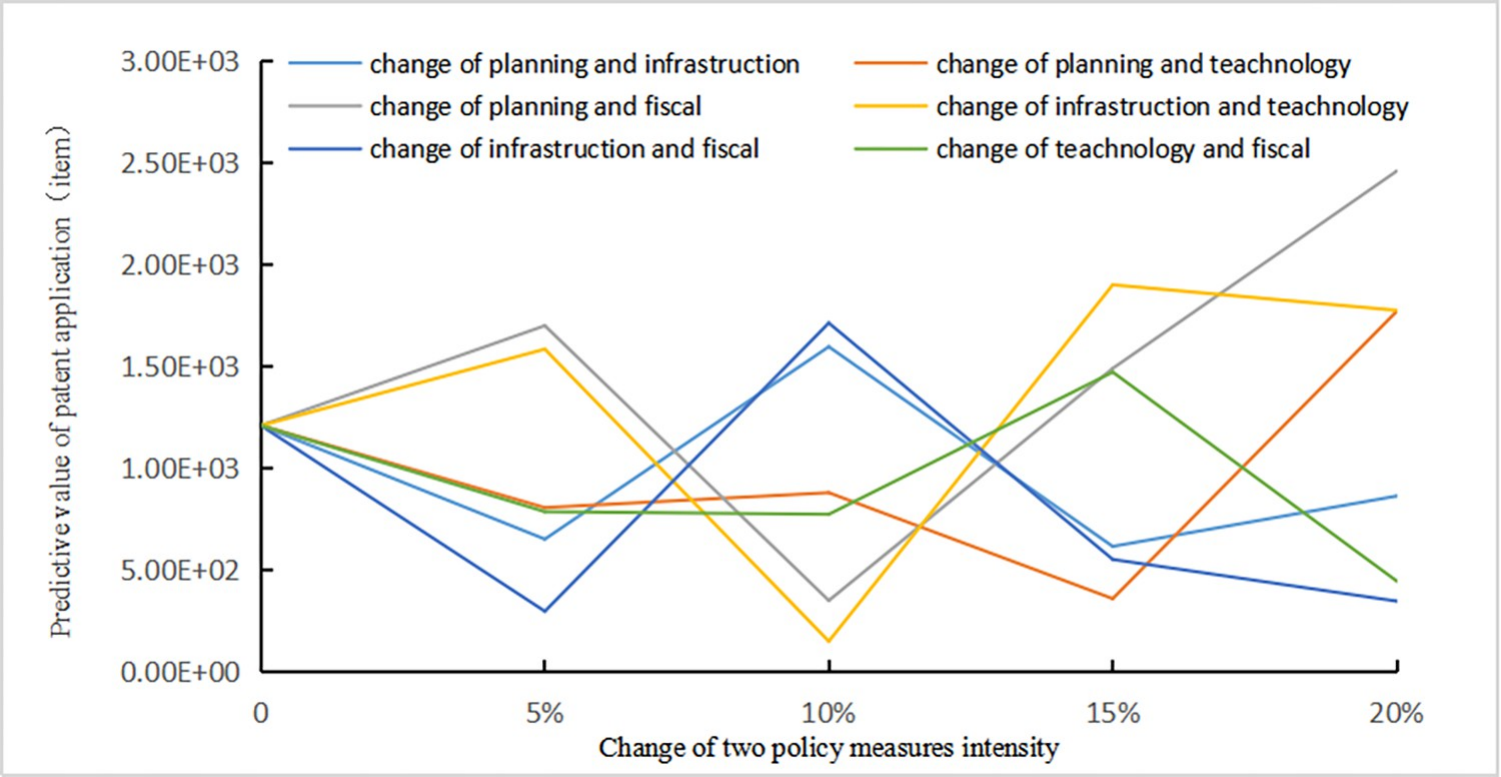
Relationship between intensity of two policy measures and number of patent applications.

According to Fig 8, it shows strong volatility and uncertainty when the intensity of two policy measures increase at the same time. The changing trend of enterprise technological innovation becomes more complex, even in a state of contradiction. When the change of policy intensity is less than 10%, the incentive effect of simultaneous change of planning and technology or technology and fiscal and tax policy intensity on technological innovation is small. When the intensity boundary is exceeded by 10%, the excitation effect increases rapidly. However, under the change of policy intensity of 5%, 10% and 15%, technological innovation of enterprises shows a certain contradiction. With the increase of intensity variation, this contradiction tends to expand. It can be seen that there is a moderate range of policy synergy in the incentive effect of technological innovation of new energy vehicle enterprises.

Scenario 2 applies to the early stage of industrial development. Various policy measures are coordinated to maximize the incentive effect, and the policy synergy mechanism is matched for enterprises in different links of the new energy automobile industry chain. For upstream raw material enterprises, the demand pull of the middle and downstream links is the main incentive, which is affected by the overall environment of industrial policy. For midstream parts enterprises, technical specifications policy should be the core, and the synergy with planning guidance and fiscal and tax policy measures should be improved. Greatly enhancing the intensity of planning guidance and technical standard policy measures can form an effective policy incentive system. For downstream terminal enterprises of vehicle and charging facilities, infrastructure policies should be taken as the core, and technical specifications and fiscal and tax policy measures should be coordinated to encourage technological innovation of enterprises.

Scenario 3: The influence rule of the technological innovation states of NEV enterprises when the intensity of three policy measures increase 5%, 10%, 15% and 20% simultaneously.

**Fig 9:**
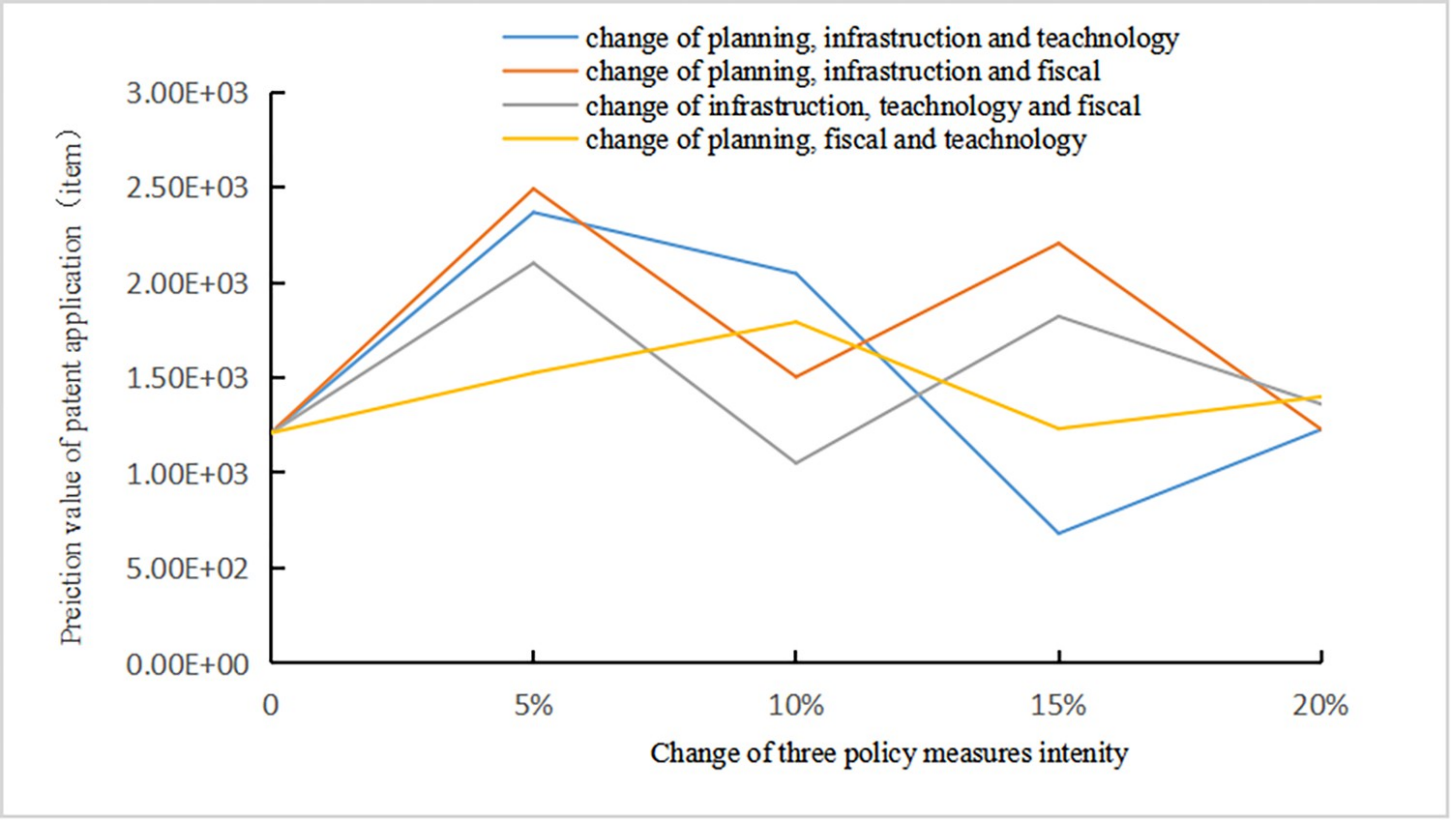
Relationship between the intensity of three policy measures and number of patent applications.

As shown in Fig 9, when the intensity of any three policy measures increase, the initial technological innovation is better than the other two scenarios. When the intensity of the three policy measures increases by 5%, the incentive direction of enterprise technological innovation is the same, but the incentive effect is different. The incentive effect is the largest when the intensity of guidance, infrastructure and fiscal and tax policy measures increase, while the incentive effect is the most continuous when the intensity of guidance, fiscal and tax and technology policy measures increase at the same time. However, with the increase of intensity, the incentive effect of enterprise technological innovation is generally tightened. The incentive effect of different policies began to show positive and negative polarization when the intensity increases to 10%. The difference of incentive effect of technological innovation is more prominent when it is increased to 15%. When the intensity increases to 20%, the technological innovation development of new energy vehicle enterprises tends to be unified, and the maximum incentive effect is weaker than the previous several stages. It can be seen that there is an optimal allocation of policy structure for the effective incentive range of new energy vehicle enterprises.

Scenario 3 applies to the middle and late period of industrial development, when the policy system has been basically formed. The change of policy synergy is the process of policy structure optimization. A small increase in the intensity of the three policy measures at the same time can effectively promote industrial technological innovation, and this policy system optimization method can reduce government expenditure to the greatest extent. On the other hand, we need to avoid excessive increase in the intensity of policy measures and excessive dependence on policy so as to successfully complete the transformation from policy-driven to market-driven of new energy vehicle industry.

## Conclusion

This paper designs an interpretable automatic coding machine and fractional momentum RBF neural network model for technology innovation prediction, and new energy vehicle policy and technology innovation of China are taken as an example to verify the validity of the model. Three experimental scenarios were designed to reveal the inherent nature and law of policy synergy and technological innovation by NEV enterprises through the number of policies changing intensity and the extent of the changes in intensity. The specific conclusions and implications are as follows.

1. The improved RBF neural network model proposed in this paper is more stable for updating weights and more flexible for adjusting parameters. Therefore, the accuracy of innovation prediction is higher. According to the simulation results of the test set data, the average relative errors of the RBF neural network model with fractional momentum, the RBF neural network model, and the BP neural network model are 7.36%, 9.46% and 10.57% respectively. The feasibility and robustness of the proposed model in this paper are verified.
2. The incentive effect on technological innovation has obvious heterogeneity under different scenarios and shows directional difference under different intensities. Different policy coordination modes should be matched in different development stages of new energy vehicle industry. The incentive effect of technological innovation of new energy vehicle enterprises can be maximized and sustained by adjusting the degree of coordination of policy measures through policy intensity. The reconstruction and optimization of the policy system to form the policy space for technological innovation is the key to achieve the high-quality development of new energy vehicles in China.

## Supporting information

S1 Data (XLS)
